# The effect of living a ‘yogic lifestyle’ on stress response and self-image in healthcare professionals: a pilot study

**DOI:** 10.2144/fsoa-2019-0154

**Published:** 2020-05-27

**Authors:** Kavita Beri, Vidya Menon, Edgardo Guzman, Claudia Chapa, Raxa Patel, Masood A Shariff, Moiz Kasubhai

**Affiliations:** 1 Department of Internal Medicine, NYC Health + Hospitals/Lincoln, 234 East 149th Street, The Bronx, NY 10451, USA; 2 BE Mind Body Skin, 3200 Sunset Ave Suite 107, Ocean, NJ 07712, USA; 3 Department of General Psychiatry, NYC Health + Hospitals/Lincoln, 234 East 149th Street, The Bronx, NY 10451, USA

**Keywords:** burnout, energy balance, healthcare, mindfulness, tantra yoga, well-being, wellness, workplace stress

## Abstract

**Background:**

Healthcare staff in modern metropolitan settings face higher rates of burnout characterized by emotional stress and difficulty coping with not only building work pressure but also balancing personal life stress. The aim of this pilot study was to see the impact of a yogic lifestyle, incorporating diet, exercise and mindfulness activities based on tantra yoga.

**Materials & methods:**

Fifteen participants were recruited and completed three or more of the interventions.

**Results:**

The 4-week pilot study showed increased self-compassion and decreased stress among the participants.

## Lay abstract

The healthcare workforce faces higher rates of burnout characterized by emotional exhaustion, depersonalization and difficulty in connecting with patients in meaningful ways. Yogic lifestyle is a wellness initiative incorporating elements of mindfulness, mindful eating and mind-body exercise. The aim of this pilot study is to evaluate the effect of yogic lifestyle on perceived stress and self-compassion among healthcare professionals. Our results show a statistical improvement in the parameters studied based on perceived stress and self-image.

Living a life with an active practice of mindfulness and engaging in yoga has been shown to improve stress response in participants. Research suggests that high levels of stress can negatively affect both physical and mental health and influence all body systems [1]. Stress is seen as an essential and accepted part of being a healthcare professional, but it has been shown to take its toll. The result is ‘burnout’, which is defined as a combination of emotional exhaustion, depersonalization and perceived inefficacy resulting from long-term job stress [2,3]. This could make it difficult to connect with patients in meaningful ways [4–6]. It can also harm the effectiveness of a healthcare professional by decreasing attention, reducing concentration and impinging on decision-making skills [7]. Practices of mindfulness have been shown to increase self-awareness and self-compassion and decrease uncertainty and rumination (a pattern of elaborated analytical thinking). These techniques include breath awareness, mindful movement (gentle yoga) and a body awareness practice. Yogic lifestyle brings into account the different variations of a healthy lifestyle combined into one practice. Mindfulness to self, mindful eating and mind-body exercise with yoga could be practiced in every part of an individual’s life, including the workplace.

There is an evidence-based literature of how mind–body medicine can improve functionality in the work environment as well as self-image. Practicing mindfulness has been linked to positive health outcomes including decreased anxiety, chronic pain and increased immune function [8–10]. An interventional approach helps manage stress called mindfulness-based stress reduction, it can be taught through practice. This technique involves a moment to moment awareness, purposefully paying attention to the present with a nonjudgmental attitude [11]. With meditation, this inherent aspect of consciousness can be enhanced. Yoga as a practice has been shown to reduce stress through the inflammatory response [12]. Practicing both mindfulness and meditation requires all of the techniques that are used within mindfulness-based stress reduction programmes. It has been shown to affect stress levels differently between practitioners and nonpractitioners [13]. Healthcare professionals (physicians, nurses and clinical staff), altogether, are prone to stress and experience its effects. Studies on mindfulness and its practices have been done on these professionals and have shown results including improvement in stress levels, self-perceived well-being and mindfulness; through standardized tests a continued practice of these skills can have an effect on oneself and on others through a herd effect [14–17].

Mindful eating is another aspect that trains an individual to bring about focus and reduce distraction pertaining to foods. It is described as a nonjudgmental awareness of physical and emotional sensations associated with eating [18]. In combination, mindful meditation and yoga with mindful eating are areas of current study and could provide a skill set to aid healthcare practitioners in dealing with stress, burnout and anxiety.

The literature is lacking in demonstrations of the practice of mindfulness and yoga and their impact on healthcare professionals who work in high stress environments. Indeed, no specific studies on the effects of incorporating mindfulness, yoga and mindful eating in healthcare professionals exists. As part of our study’s objective, we conducted a pilot prospective study comprising a 4-week program of instructional sessions incorporating mind–body exercises (i.e., meditation and yoga) and mindful dieting, for healthcare, clinical and administrative, participants. The purpose of this study was to evaluate the efficacy of improved stress response and perceived self-image. With the growing awareness of burnout in healthcare professionals, the promotion of well-being in physicians has been the focus of several recent initiatives, at least within our institution. In this current study, improvement in stress response and self-image are evaluated following the introduction, practice and reinforcement of mindfulness, mindful eating and yoga practices in healthcare, clinical and administrative, participants.

## Materials & methods

Participants were recruited from a single metropolitan healthcare facility allowing healthcare staff, clerical workers and volunteers to participate in this prospectively designed single-group pilot study. The study was reviewed and approved by the Institutional Review Board (IRB) and assessed for safety and feasibility (IRB#18-001). All the participants signed informed consent. Recruitment was performed by advertising through in-hospital circulating flyers and newsletters, and in-house email blasts, and the study was introduced at administrative and clinical staff meetings that comprised of nurses, physicians and clerical staff. The study was open from November 2018 to December 2018 for recruitment and the study period was from January 2019 to February 2019.

Inclusion criteria for participants of both genders were: age of 18 years or above and healthcare staff working the day shift in the hospital. Excluded were participants already practicing mind–body medicine or complementary and alternative medicine programs, pregnant females, physical limitations affecting practice of yoga or inability to participate in the activities due to conflict or interruption of patient care. No previous experience and knowledge of the mindfulness or yoga techniques was required for participation.

### Procedures

The program consisted of three sections: mindfulness, mindful eating lessons and yoga philosophy with yoga meditation movement classes, once a week for 1.5 h per week in group sessions for 4 weeks.

## Mindfulness

Practice of mindfulness was achieved by providing an orientation on ‘what is mindfulness?’ and the steps for practicing it. Research team members provided information on mindfulness, awareness, breathing techniques, orienting thoughts positively and bringing focus to the ‘present’. These techniques were elaborated on and practiced in the yoga sessions that followed. The participants were provided with Mindfulness Online Podcasts developed by UCLA Mindful Awareness Research Center that provided a course on applying mindfulness and its aspects through audio records [19] as a part of the mindfulness sessions to be used throughout the duration of study. Participants were encouraged to listen to these podcasts at home to practice mindful meditation, breathing techniques and other basics of meditation. The podcast link was emailed to all the participants. After the orientation session at week 1, reminders were given at the beginning of the other sessions at week 2, 3 and 4 to take time and go through the audio records on the website.

## Mindful eating

The participants had an initial mindful eating orientation session (week 1) in which they were taught the practices and incorporation of mindful eating during their daily routines. Instruction included an ideal plate guideline with instructions on eating while limiting distractions, placing focus on texture and colors and utilizing the senses to smell, taste and focus. These instructions were provided as a hand-out to all participants and they were encouraged to apply these practices throughout the week. Reminders were given as prompts during the beginning of the consecutive sessions (week 2, 3, 4) with referral to utilization of the hand-out for review. Below is an excerpt mentioned at the beginning of each session:

Mindful eating practice starts with – sit comfortably. Take 3 slow deep breaths. Bring your attention to your food. Experience what it's like to eat your food, use all your senses to experience it. Before and as you eat notice the look, smell, texture, sounds, and taste of your food. As you are mindfully eating you may be distracted by thoughts or other things. Just notice them without judgement and let them go, turning your attention back to what it's like to be eating.

## Yoga exercises

In the yoga sessions, the participants were taught theoretical aspects of yoga philosophy and the concept of the ‘energetic body’ maps (chakras). The yoga sequence was based on tantra yoga, which included a basic sequence of sun salutation (surya namaskar) with cued visualizations. There were guided meditation movements that included yogic breathing (pranayama) and sacred geometry visualization. The yoga and meditation practices were held in an auditorium reserved for the study with doors that closed at the hospital and room to spread out. The session was led by a trained YOGA/Meditation Certified Instructor. Participants were encouraged to bring their own yoga mats; however, the research team provided mats if needed. Loose-fitted clothing was recommended for the sessions. The participants were encouraged to continue a daily set practice of the yoga sequences taught.

The education on yoga philosophy was based on the yoga sutras and the anatomy of the energetic body based on tantra yoga philosophy; Supplementary Table 1 shows a chart discussed with students regarding the energetic centers. The yoga sequence was based on energy flow and visualizations based on the chakras.

The yoga session took place once a week for an hour and progressed over the course of the study. Preplanned variations of asanas were implemented to meet the capabilities of participants. Participants were provided with instructions to practice the yoga poses during the remainder of the week. Online educational material, with videos and blogs were provided to the participants to encourage a home practice routine daily.

## Outcome measures

The questionnaire utilized in the study comprised validated, self-reporting inventories: the Perceived Stress Scale 10-question (PSS) and the Self Compassion Scale (SCS). All participants were given the two questionnaires to be filled out prior to the starting of any intervention and were again given them at the completion of the 4-week duration of the course along with a poststudy questionnaire that recounted the frequency of independent session (mindfulness, mindful eating, yoga) practiced by the participants.

The PSS reports an individual’s experience of stress. The tool measures psychological manifestations of stress and is designed to assess the degree to which individuals appraise situations in their life as stressful. Responses are on a 5-point Likert scale ranging from 0 (never) to 4 (very often). Four of the positively stated items were reverse. Results were reviewed as sum total and categorized (low = 0–13; moderate = 14–26; high = 27–40) [20,21].

**Figure 1.**
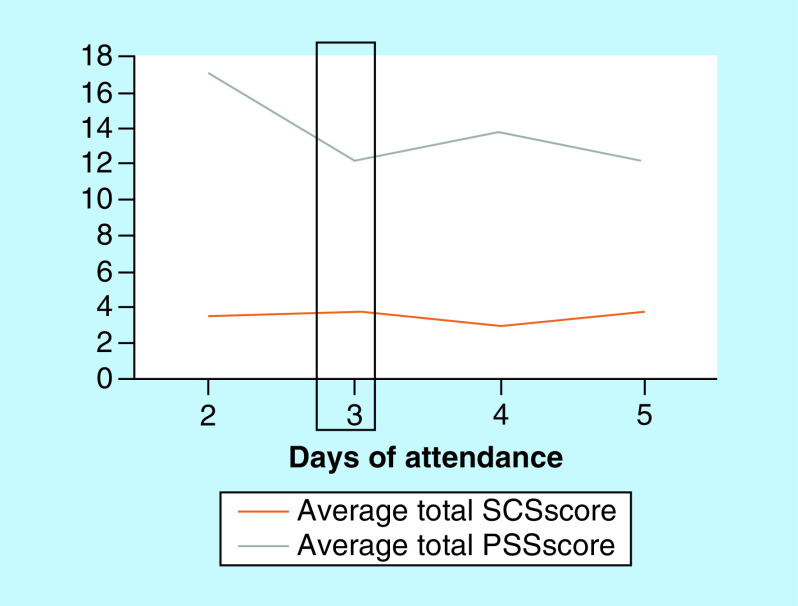
Distribution of perceived stress scale and self-compassion scores based on days of participant attendance (PSS; SCS). PSS: Perceived stress scale; SCS: Self-compassion scores.

The SCS survey has 26-items that measure components of self-compassion using a 5-point Likert scale ranging from 1 (almost never) to 5 (almost always). The SCS measures self-compassion through six subscales classified as self-kindness, self-judgment, common humanity, isolation, mindfulness and over-identification [22]. Three of the scales are reverse scored: self-judgment, isolation and over-identification. The mean scores from the subscales were combined to yield a total score, which reflects a global measure of self-compassion. Higher scores indicate greater self-compassion. Results were reviewed as means and categorized (low = 1–2.5; moderate = 2.5–3.5; high = 3.5–5.0). A graphical representation was generated to assess the trend of the PSS and SCS scores plotted over the days of participant attendance (Figure 1). The two scales were emailed to the participants by their participant number and they were requested to complete and drop them off into a sealed box.

Hedges’ g effect sizes were calculated to determine the magnitude of differences between trial pairings. The likelihood that the true value of the effect represented a worthwhile change was assessed using the following thresholds: trivial (<0.2), small (0.2–0.5), moderate (0.5–0.8) and large (>0.8) for Hedges’ g and are reported by mean differences and 95% CIs.

Paired outcomes for each participant were distributed and tested using t-test, Fisher’s exact test and chi-square tests were performed to assess statistical differences between participants pre and postintervention scores. All data analyses were performed using GraphPad Prism (GraphPad Software, Inc., CA, USA) and Microsoft Excel (Microsoft Research Lab,WA, USA).

## Results

Fifteen of the 21 participants who volunteered were retained for the duration of the study. The other six participants were not able to attend all the sessions or complete the surveys because of scheduling issues, emergent patient care needs and were not included in the analysis. The mean age of the participants was 38 years (standard deviation [SD]: 11), with a range of 26–68 years and 11 participants were female (79%). The majority were healthcare professionals (60%) with eight of the participants as resident physicians (53%), one healthcare specialist (6%) and remainder comprised of hospital’s administrative staff. Patient characteristics are listed in Table 1.

**Table 1.** Participant characteristics (n = 15).

Characteristics	Statistics
– Mean ± SD	37.9 ± 11.5
– Median (range)	34 (26–68)
Age group, years	
– <30	2 (13.3%)
– 30–40	9 (60.0%)
– >41	3 (20.0%)
– No response	1 (6.7%)
Gender	
– Male	4 (26.7%)
– Female	11 (73.3%)
Departmental titles	
– Healthcare professionals	8 (53.0%)
– Healthcare specialist	1 (7.0%)
– Administrative staff	6 (40.0%)
Interventions attended, sessions	
– 2	1 (6.7%)
– 3	3 (20.0%)
– 4	11 (73.3%)

Data is presented as mean and SD for continuous variables and frequency-percent for categorical variables.

SD: Standard deviation.

During the 4-week study period, participant’s PSS scores decreased while their total SCC scores increased (Table 2). The PSS score decreased by 7 points (pre-19.9 ± 5.3 vs post-13.3 ± 4.3; p = 0.002), a 33% difference from baseline to postintervention. The SCS score increased by 17% between the baseline and postintervention (2.93 ± 0.73 vs 3.42 ± 0.66, respectively; p = 0.0004). Categorical changes in pre and postintervention data from PSS and SCS were not significant. In PSS, there were no participants that scored in the high perceived stress range (≥27 total points), whereas there was a decrease in the moderate perceived stress category (14–26 total points) of 27% (4 participants) that shifted to the low perceived stress range (≤13 total points); a total of 4 participants made a shift from the moderate to low category. No participants were found to be in the high category for perceived stress preintervention. Figure 1 was generated to assess the trend of the survey scores over the days of participant attendance and estimated at day 3 the scores predicted a change in PSS score that decreased and no change in the SCS score.

**Table 2.** Outcomes from preintervention compared with postintervention by perceived stress scale and self-compassion score surveys.

Scale	Preinterventional phase	Postinterventional phase	p-value
Perceived stress scale score	15.3 ± 3.56	13.3 ± 4.33	0.0104
– Hedges’ g	0.5046 (-2.0 [-3.45–0.55])		
PSS stress categories			0.2148
– Low	2 (13%)	6 (40%)	
– Moderate	13 (87%)	9 (60%)	
– High	0	0	
Self-compassion score	2.93 ± 0.74	3.42 ± 0.66	0.0004
– Hedges’ g	0.419 (0.286 [0.133 − 0.438])		
SCS subscales			
– Self-kindness	2.93 ± 0.79	3.52 ± 0.72	0.0032
– Self-judgment	2.80 ± 0.88	3.29 ± 0.78	0.0083
– Common humanity	3.20 ± 0.72	3.53 ± 0.66	0.0156
– Isolation	2.82 ± 1.19	3.30 ± 1.03	0.0344
– Mindfulness	3.25 ± 0.77	3.58 ± 0.61	0.0069
– Over-identified	2.55 ± 0.91	3.28 ± 0.95	0.0008
SCS categories			0.4843
– Low	3 (20%)	2 (13%)	
– Moderate	9 (60%)	7 (47%)	
– High	3 (20%)	6 (40%)	

Data is presented as mean and SD for continuous variables and frequency-percent for categorical variables. Hedges’ g is presented with mean difference (95% CI).

PSS: Perceived stress scale; SCS: Self-compassion scores; SD: Standard deviation.

For SCS categorical change, there were 3 participants with a low self-compassion score at baseline and that changed to 2 participants; for high self-compassion score there was a 20% increase (3 participants to 6 postintervention); a total of 4 participants made a shift from a lower category to a higher one (3 Moderate to High & 1 Low to Moderate) postintervention.

The SCS subscale scores increased and were significant for: self-kindness (difference [diff]-0.5; p = 0.003); self-judgement (diff-0.49; p = 0.008); common humanity (diff-0.33; p = 0.015); isolation (diff-0.48; p = 0.034); mindfulness (diff-0.33; p = 0.006); and over-identification (diff-0.73; p = 0.0008). The largest increase was seen in the score for over-identification with thoughts and feelings, which is a reversed category giving the theme a positive spin with ‘not being overidentifying’.

Subgroup analysis was also performed for gender differences in the cohort for the scoring of PSS and SCS. There were more females (73%) than males (27%) in our sample size. PSS scoring was significantly higher for females (pre-16.5 ± 1.81 vs post-14.3 ± 2.80; p = 0.042) than males (pre-12.0 ± 5.35 vs post-10.3 ± 6.70; p = 0.102). SCS scoring was again significant for females (pre-2.75 ± 0.64 vs post-3.38 ± 0.60; p = 0.0002) compared with males (pre-3.40 ± 0.88 vs post-3.52 ± 0.91; p = 0.409); categorically males did not change from their initial ranking to a higher one, but females did (high: 1 to 4, moderate: 8 to 6 and low: 2 to 1, pre to postintervention, respectively).

Effect size was calculated using the Hedges’ g and presented a moderate effect for preintervention compared with postintervention for PSS (mean difference [95% CI] = -2.0 [-3.45–0.55]; Hedges’ g = 0.5046) and SCS (mean difference [95% CI] = 0.286 [0.133–0.438]; Hedges’ g = 0.419) scales.

Table 3 reports the results of the follow-up self-reported compliance questionnaire sent to the participants. Self-reported compliance of 4–5-times/week with practice of yoga was performed 40% of the time, mindful eating practiced 47% and listening to mindfulness audio 2–3-times/week was 40%.

**Table 3.** Self-reported compliance study interventions.

How often…listen to the MINDFULNESS audio links during the week?	Results
0–1	6 (40%)
2–3	6 (40%)
4–5	1 (7%)
≥6	2 (13%)
How often…practice MINDFUL EATING during the week?	
0–1	4 (27%)
2–3	4 (27%)
4–5	7 (47%)
≥6	0
How many days…practice YOGA during the week?	
0–1	3 (20%)
2–3	5 (33%)
4–5	6 (40%)
≥6	1 (7%)

## Discussion

The objective of this study was to evaluate the efficacy of the practice of mindfulness, mindful eating and yoga exercises in participants that belonged to a metropolitan healthcare facility concerning their perceived stress and self-compassion. Healthcare professionals engage in a high-level stress environment, especially in a metropolitan health system, thus this project was a pilot study in developing an overall well-being initiative to aid and provide skills in understanding and overcoming stressors. The participants had an overall decrease in perceived stress score and an increase in self-compassion score that were significant after the duration of 4-weeks. We also noticed a gender difference present in our participants, with an overall low perceived stress score for males at baseline, but with a greater difference in change for the females after the 4-week duration. Self-compassion score also showed a gender difference with increased scores in females, preintervention compared with postintervention, whereas males did not show a significant change. The attendance to the study sessions and survey scores, when extrapolated, showed a change after presenting to three session of the study interventions.

In January 2001, the Joint Commission on Accreditation of Healthcare Organizations mandated all hospitals to have a process to address physician’s well-being [6]. Despite this, physicians continued to feel dissatisfied and distressed [23]. Given the negative impact this can have on physicians and their families, as well as on patients and healthcare organizations, current studies are attempting to address the well-being of healthcare professionals.

The holistic practice of yoga, its teachings and meditation have been the foundations of modern-day mindfulness concepts. Ancient philosophical texts have discussed the presence of a multidimensional state of the body, with its energetic and emotional layers that can be healed by certain yoga asanas, lifestyle and meditation techniques [24]. Tantra yoga sequences and philosophical teachings used in the study focused on sequences that helped align the energetic centers (chakras) and helped balance the flow of ‘prana’ (life force) through those centers to balance the emotional states [25,26]. The concept of energy healing of the body falls under the umbrella of ‘vibrational medicine’ and the concept of biofield science [24].

Although mindfulness meditation has an ancient origin, it has been adapted to Western contexts to treat patients with a diversity of physical and psychological conditions. The Western definition of mindfulness has yet to be agreed upon by scientists; however, the most commonly used one belongs to Professor Jon Kabat-Zinn, who translates it as the awareness that emerges through paying attention in a particular way, on purpose, in the present moment and nonjudgmentally [11,27]. It is conceptualized as a state in which one is aware of the present moment, acknowledging and accepting it. In addition, it allows for the ability to disengage from maladaptive patterns of the mind that would make one vulnerable to stress responses [8]. It is further believed that excessive orientation toward the past or future when dealing with stressors can relate to feelings of depression and anxiety [28]. Mindfulness thus allows one to cope with stress in a healthier, more effective way [27].

Mindfulness-based stress reduction is an educational program created for medical patients to relieve stress, pain and illness [27]. The key components include: sitting meditation involving awareness of body sensations, thoughts and emotions while returning the focus of attention to the breath; hatha yoga, which consists of postures and stretches designed to enhance greater awareness of and to balance and strengthen the musculoskeletal system; and body scanning, which is a progressive movement of attention through the body from head to toes [9]. These practices were observed in several small randomized trials, one with patients undergoing ultraviolet phototherapy for severe psoriasis (a psychological stress) and the other a worksite intervention to company employees who were monitored by quantitative electroencephalogram (EEG) and immune responsivity. Respectively, guided mindfulness meditation and a mindfulness-based stress reduction program were used for the two trials with clinical effectiveness (i.e., rapid skin clearing in the psoriasis patients and positive emotional expression on EEG in company employees) [28]. With different mindfulness interventions, different classes of individuals with different life situations in different environments can benefit from its effectiveness.

In the current era of microbiome science, plant-based nutrition has been shown to be an important driver of stabilizing the gut microbiome. There is an established connection to improved states of mental well-being through the presence of a gut-brain axis in the microbiome–host interaction. This complex interaction is an interplay of neuroendocrine and neurohumoral pathways that communicate and connect mental and physiological states. Yogic lifestyle includes plant-based substitution and eating in a mindful way in order to help heal and achieve a state of well-being emotionally [29,30].

Limitations included a small sample size and short duration of follow-up. Further study limitations include lack of randomization and a control group. We also acknowledge there is a limitation with age groups that were recruited, as we opened the study for all comers, rather than focusing on one group such as residents; we mixed the sessions with clinical and nonclinical staff, which caused the age variation. However, considering that the work environment as a constant in a community metropolitan hospital, the perceived stress scales measured were based on the work environment being constant for each participant. Future studies may be designed as a two-arm evaluation with a control group and, in addition, a diary to track actual practice could be beneficial in quantifying the overall effect of each intervention. Our institution has taken the lead with this pilot study to create an ongoing Wellness Initiative that compiles all the elements discussed in this study.

## Conclusion

The lifestyle approach of yoga, explained by the concept of ‘Yama and Niyama’ in the ancient yogic text of the yoga sutras, is to guide a stronger state of mental well-being. Breathing techniques and postures that help the flow of vital energy create a sense of well-being, not just emotionally, but also physiologically by improving immune status and antioxidant levels in the body that help restore, heal and regenerate. The connection of visualization during meditation and focus on balancing the energy centers in oneself, extrapolates the connectivity of emotional balance related to energetic harmony within the body. Healthcare being high in the rank of stressful career paths, a state of mental clarity and emotional balance could lead to satisfaction in both personal and professional betterment of interpersonal relationships and doctor–patient interactions.

## Future perspective

The implementation of yogic lifestyle and mindfulness practices in the field of healthcare can help foster a strong work environment and also help in better delivery of care by the improved emotional and balanced self-image in the practitioners. Large-scale studies are needed to understand in detail the impact and changes in the emotional states and the impact of duration of practice. Getting a retrospective analysis of the participants 6-months poststudy would be interesting in order to understand compliance and long-term impact of implantation of this lifestyle.

**Executive summary**

- This pilot study comprised a mindfulness initiative of a yogic lifestyle undertaken in response to higher physician rates of stress and burnout.
- It aimed to determine the efficacy of following a yogic lifestyle protocol on overall self-image and stress response.
- A 4-week protocol including meditation, practice of tantra yoga, diet and lifestyle education was given to participants.
- The study demonstrated improvement in the stress response and self-image of the participants.
