# Supplementary material for: The effect of living a ‘yogic lifestyle’ on stress response and self-image in healthcare professionals: a pilot study
Source: Future Sci OA. 2020 May 27;6(6):FSO473. doi: 10.2144/fsoa-2019-0154 (PMC7351084; doi:10.2144/fsoa-2019-0154)
Supplement: Supplementary file 1 [file fsoa-06-473-s1.docx]

Supplementary material

Supplementary Table 1: Chakras connected to the Endocrine Physiology and Emotional Body (Courtesy BE SCHOOL OF LIGHT)

| Chakra/ Energy Center | Endocrine Glands | Balanced Emotional Body |
| --- | --- | --- |

| Crown Chakra  (Sahasrara) | Anterior pituitary, Cerebral cortex, Cerebellum | Connected to universal source, unity consciousness, seeing a sense of connection and oneness in all beings |
| --- | --- | --- |
| Third Eye  (Ajna) | Pineal gland, Cerebellum | Intuition, trusting yourself,  Higher perspective and outlook towards daily situations. Clairvoyance, Clairaudience |
| Throat Chakra  (Vishuda) | Thyroid, Parathyroid | Being able to speak your truth with confidence, and live authentic |
| Heart Chakra  Anahata | Adrenal cortex, Thyroid, Anterior pituitary | Unconditional love, and feeling secure about self-worth and love for others |
| Solar Plexus Chakra  Manipura | Pancreas, Liver, Adrenal Medulla, GI tract | Allowing balanced transformation and changes in life without resistance, balanced self-esteem. |
| Sacral Chakra  Swadhisthana | Ovaries, testicular glands, | Creative inspiration and improved self-esteem improved interpersonal relationships. |
| Root Chakra  (Muladhara) | Prostrate, Lower Gi Tract, | Feeling grounded, connected, self confidence |
